# The combination of multiple plant growth promotion and hydrolytic enzyme producing rhizobacteria and their effect on Jerusalem artichoke growth improvement

**DOI:** 10.1038/s41598-023-33099-x

**Published:** 2023-04-11

**Authors:** Natthawat Sritongon, Sophon Boonlue, Wiyada Mongkolthanaruk, Sanun Jogloy, Nuntavun Riddech

**Affiliations:** 1 Department of Microbiology, Faculty of Science, Khon Kaen University, Khon Kaen, 40002 Thailand; 2 Department of Agronomy, Faculty of Agriculture, Khon Kaen University, Khon Kaen, 40002 Thailand

**Keywords:** Microbiology, Environmental sciences

## Abstract

Rhizobacteria are well recognized for their beneficial multifunctions as key promoters of plant development, suppressing pathogens, and improving soil health. In this study, experiments focused on characterizing the plant growth promotion (PGP) and extracellular hydrolase production traits of rhizobacteria, and their impact on Jerusalem artichoke growth. A total of 50 isolates proved capable of either direct PGP or hydrolase-producing traits. Two promising strains (*Enterobacter* *cloacae* S81 and *Pseudomonas* *azotoformans *C2-114) showed potential on phosphate and potassium solubilization, IAA production, and 1-aminocyclopropane-1-carboxylic acid deaminase activity and hydrolase production. A hydrolase-producing strain (*Bacillus* *subtilis* S42) was able to generate cellulase, protease, amylase, β-glucosidase, and phosphatase. These three selected strains also gave positive results for indirect PGP traits such as siderophore, ammonia, oxalate oxidase, polyamine, exopolysaccharide, biofilm, motility, and tolerance to salinity and drought stress. Colonization was observed using a scanning electron microscope and rhizobacteria appeared at the root surface. Interestingly, inoculation with consortia strains (S42, S81, and C2-114) significantly increased all plant parameters, including height, biomass, root (length, surface, diameter, and volume), and tuber fresh weight. Therefore, we recommend that potential consortia of PGP and hydrolase-producing rhizobacteria be employed as a biofertilizer to improve soil and boost crop productivity.

## Introduction

Jerusalem artichoke, commonly known as sunchoke (*Helianthus* *tuberosus* L.), is one of the most profitable crops due to the wide application of its tuber^1,2^. The tuber is rich in inulin, a type of prebiotic that promotes the growth of beneficial microorganisms in the colon and lowers cholesterol, blood sugar, and the risk of colon inflammation in both humans and animals^3^. In the bioethanol industry, sunchoke tuber is considered a potential alternative material for ethanol production, similar to corn grain, sugarcane, and cassava, due to its rapid harvesting^4^. In addition, bioactive substances found in the leaves and flowers function as antibiotics, anti-inflammatories, and antioxidants^1^. The plant has significant implications for the agricultural, food production, bioenergy, and pharmaceutical sectors. In Thailand, several genotypes were successfully developed in suitable plantations for high yield^5,6^. However, the high productivity of this crop is promoted by the utilization of high doses of synthetic fertilizers^1^. The use of chemical fertilizer causes nutrient imbalance in the soils and leads to environmental pollution^1,7^. Therefore, it is crucial to achieve sustainable agricultural practices and protect the ecosystem from harm.

The application of useful microbes is an environmentally friendly method that facilitates crop productivity and soil maintenance. Beneficial microbes have two strategies for direct and indirect promotion of plant productivity, respectively plant growth promotion (PGP) and phytopathogen suppression^8^. Plant growth promoting microorganisms (PGPM) play a role in enhancing plant growth with several recognized traits: fixing nitrogen gas; providing soluble inorganic forms of phosphate, potassium, zine, and silicon; and synthesizing plant hormones such as cytokinin, gibberellin, and indole-3-acetic acid (IAA)^8^ that are strong effects on root surface and elongation^9^. PGPM can produce 1-aminocyclopropane-1-carboxylic acid (ACC) deaminase activity which diminishes ethylene levels, which helps plants in tolerating biotic and abiotic stressors^10^. Moreover, various indirect mechanisms, including sulfur oxidation and production of bacteria-to-plant signal molecules (i.e., oligosaccharides, peptides), contribute to the growth promoting traits of PGP rhizobacteria on plants^11^. Hydrolase released by the PGP rhizobacteria has multifaceted roles; it not only suppresses pathogens, but also degrades organic matter and circulates soil nutrients in the rhizosphere zone^12–14^. Hydrolases such as cellulase, protease, amylase from bacteria are more effective catalysts^12,13^. Cellulase, amylase, and β-glucosidase are linked to rotate of cycling organic carbon, which is used by plants and microorganisms as a source of nourishment. The cellulase enzyme transforms cellulose in organic matter into cellobiose, glucose, and oligosaccharide^15^. Amylase converts starch to oligosaccharides and maltose, whereas β-glucosidase converts cellobiose to glucose. β-glucosidase and phosphatase are employed as soil quality indicators^15^. Soil health may depend on these functions expressed from the abilities of microbes in soil. Therefore, it is important to find a novel PGP bacteria which can be used as a bioinoculant with multiple traits for potential inoculants in development of improving plant growth, biocontrol, and soil restoration.

A few studies have described the potential of PGP microbe isolates from sunchoke tissue and rhizosphere soil. For example, effective endophytic *Bacillus* sp. stimulated sunchoke in growth and yield in limited water cultivation^3,16^. *Exserohilum* *rostratum*, a novel effective fungal endophyte was also reported by Khaekhum et al.^2^. Suebrasri et al.^17^ discovered that novel endophytes with fungicidal metabolites suppress sclerotium disease. Therefore, the rhizosphere and tissues of sunchoke serve as a pool of beneficial microorganisms for identifying prospective strains extensively applicable to crop productivity. Therefore, we categorized effective strains performing the following functions: (i) produce PGP traits in-vitro and/or hydrolase production; (ii) colonize sunchoke roots; (iii) enhance Jerusalem artichoke growth. To our knowledge, there have been no previous reports on hydrolase enzyme activities of rhizobacteria isolated from Jerusalem artichoke and their growth promotion effects. This study aimed to characterize rhizobacteria on potential plant growth promotion and hydrolytic enzyme production activities. Samples were isolated from the rhizosphere of Jerusalem artichoke variety HEL65 and evaluated in a pot experiment for a possible synergistic effect of PGP rhizobacteria and hydrolytic enzyme-producing rhizobacteria in enhancing the growth of Jerusalem artichoke. We hypothesized that the application of PGP rhizobacteria with multifunctional activities might be able to have positive impacts on plant growth.

## Results

### Plant growth-promoting and hydrolase producing traits

A total of 50 rhizobacteria consisting of 23 isolates were screened from soil extracted medium and 27 isolates were isolated from R2A medium. They each displayed at least one PGP trait or extracellular enzyme-producing trait (shown in Table 1). Twenty-six isolates (52%) produced ACC deaminase, whereas 14 isolates (28%) showed IAA production. Solubilization of phosphate and potassium was found in 22 (44%) and 24 (48%) of the isolates, respectively. Regarding the hydrolase-producing test, our findings revealed that the majority of isolates (56%) released β-glucosidase. Twenty-two isolates or 44% possessed cellulolytic and proteolytic enzymes with a clear zone. *Pseudomonas* *azotoformans* C2-114 revealed β-glucosidase and urease activities.

**Table 1:** Characteristics of PGP and hydrolase traits exhibited by rhizobacterial isolates.

Isolates	Plant growth promoting traits					Extracellular enzyme production					
	N	Solubilization		Production		Cellulase	Protease	Amylase	β-Glucosidase	Phosphatase	Urease
		P	K	IAA	ACC deaminase						
S11	−	+	−	−	+	6.3 ± 0.3 d	2.5 ± 0.1 h–k	−	+	−	+
S12	−	−	−	−	−	8.8 ± 1.9 c	3.6 ± 0.4 d–f	1.4 ± 0.2 e	+	+	−
S14	−	−	+	−	+	−	−	−	−	−	−
S15	+	+	+	−	+	−	−	−	−	−	−
S17	−	−	−	−	−	2.0 ± 0.5 ij	−	−	+	−	−
S19	−	−	−	−	−	−	3.4 ± 0.3 ef	−	−	+	−
S21	+	−	−	−	+	−	2.9 ± 0.2 gh	−	+	+	−
S24	−	+	+	−	+	8.3 ± 0.3 c	2.7 ± 0.0 hi	−	+	−	+
S25	−	+	−	−	+	7.8 ± 0.3 c	2.4 ± 0.2 j–l	−	+	−	+
S33	−	−	+	+	+	−	−	−	+	−	−
S34	+	+	−	−	+	2.8 ± 1.0 h–j	−	−	−	−	−
S41	−	+	−	−	−	−	2.2 ± 0.4 j–l	−	−	+	−
S42	−	−	−	−	−	27.0 ± 1.0 a	5.7 ± 0.1 a	2.1 ± 0.1 cd	+	+	−
S43	+	−	+	−	+	−	−	−	−	−	−
S44	−	−	+	−	+	−	2.0 ± 0.1 l	−	+	+	−
S51	−	−	−	+	+	5.3 ± 0.3 de	3.2 ± 0.5 fg	1.7 ± 0.0 de	+	+	−
S71	−	−	−	−	−	5.3 ± 0.3 de	4.3 ± 0.1 bc	2.7 ± 0.5 ab	+	−	−
S73	−	−	−	−	−	5.3 ± 0.3 de	−	1.4 ± 0.3 e	+	+	−
S74	+	−	−	−	−	5.0 ± 0.9 d–f	−	−	+		
S81	+	+	+	+	+	−	−	−	−	−	−
S82	−	+	+	−	+	6.2 ± 0.3 d	4.6 ± 0.3 b	2.5 ± 0.2 bc	+	−	−
S83	+	−	−	−	+	−	−	−	+	−	−
S84	−	−	−	−	−	6.2 ± 1.0 d	2.6 ± 0.2 h–j	1.9 ± 0.5 de	−	−	−
R12	−	−	+	−	+	−	−	−	+	−	−
R13	−	−	−	−	−	−	3.4 ± 0.2 ef	−	+	+	−
R14	−	−	−	−	−	−	3.8 ± 0.5 de	−	+	+	−
R15	+	+	+	+	+	−	−	−	++	−	−
R16	−	+	+	+	+	−	−	−	++	−	−
R17	−	+	−	+	+	−	−	−	++	−	+
R21	−	+	−	−	−	10.8 ± 0.3 b	3.2 ± 0.5 fg	2.0 ± 0.1 de	−	−	−
R22	+	+	−	+	−	1.6 ± 0.4 j	−	−	−	−	−
R24	−	+	+	+	+	−	−	−	+	−	−
R31	−	+	+	−	−	−	−	−	−	−	+
R32	−	−	+	+	−	−	−	−	−	−	−
R33	−	+	+	−	−	−	−	−	−	−	+
R34	−	−	+	−	−	−	−	−	−	−	−
R35	−	+	−	−	+	4.3 ± 0.6 e–g	4.0 ± 0.3 cd	3.3 ± 0.5 a	+	−	−
R37	−	−	−	−	−	3.7 ± 0.3 f–h	2.0 ± 0.0 l	−	++	+	−
R38	−	−	+	+	−	−	−	−	+	−	−
R41	−	+	−	−	+	−	−	−	−	−	−
R42	+	−	+	+	+	3.3 ± 1.2 g–i	−	−	−	−	−
R43	−	−	+	+	+	−	−	−	−	−	−
R44	+	−	+	−	−	2.0 ± 0.5 ij	−	−	−	−	−
R61	−	−	−	−	−	3.8 ± 0.8 f–h	2.8 ± 0.5 g–i	−	++	−	−
R62	−	−	−	−	−	−	−	−	++	−	−
R71	+	−	+	+	+	−	2.1 ± 0.4 kl	−	+	−	−
R72	+	+	+	+	+	2.1 ± 0.3 ij	−	−	−	−	−
R81	−	+	−	−	−	5.9 ± 1.8 d	2.3 ± 0.2 i–l	2.7 ± 0.4 b	−	−	−
R83	+	+	+	−	+	−	2.0 ± 0.2 l	−	−	−	−
R84	+	+	+	−	−	−	−	−	−	−	−
C2-114	+	+	+	+	+	−	−	−	+	−	+

*N* nitrogen fixation, *P* phosphate, *K* potassium, *IAA* indole-3-acetic acid, *ACC* 1-aminocyclopropane-1-carboxylic acid, − negative result, + positive result; Production of β*-*glucosidase: + halo below 1 cm, ++ halo greater 1 cm; Production of cellulase, protease, and amylase presented as relative index value with standard deviation. Different letters represent significant differences (*p* < 0.05) according to the LSD test.

The results showed that isolates S81, R15, and R72 displayed all direct PGP traits, including nitrogen fixation, phosphate, and potassium solubilization, IAA production, and ACC deaminase. The PGP isolates also revealed the activity of one or two hydrolases. According to the results of hydrolase-producing activity, three isolates, S12, S42, and S51, exhibited multiple enzyme production traits which included cellulase, protease, amylase, β-glucosidase, and phosphatase. We selected S42 for further study due to it having significantly the highest levels of cellulase and amylase activities, compared to the other isolates.

### PGP activity

**Figure 1:**
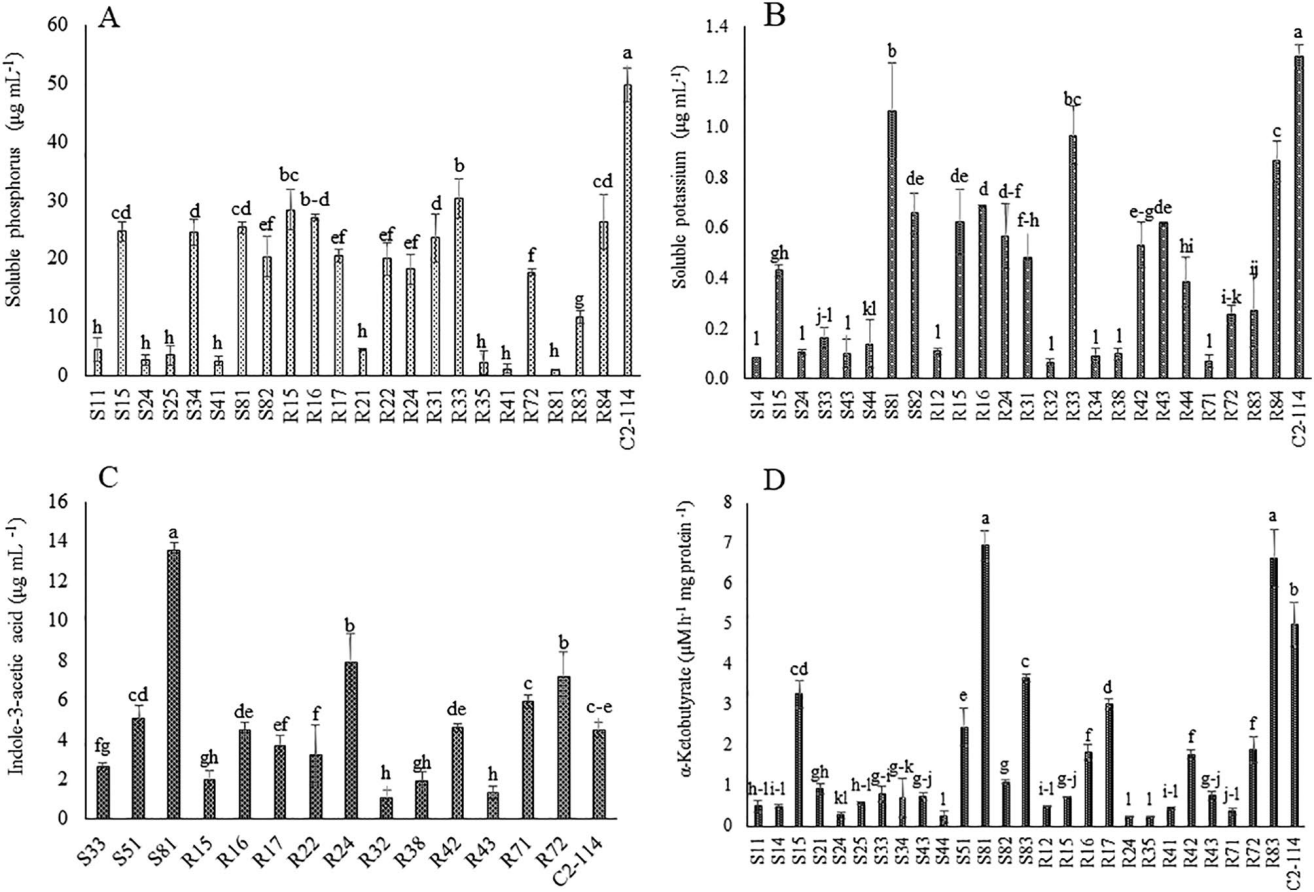
Quantitation of PGP activities including phosphate solubilization (**A**), potassium solubilization (**B**), indole-3-acetic acid production (**C**), and ACC-deaminase (**D**) of PGP isolates. Different letters on the bars represent significant differences (*p* < 0.05) according to the LSD test.

Rhizobacterial isolates obtaining PGP activities and *P.* *azotoformans *C2-114 that were used in this study and performed the quantitative assay of PGP activities. It was found that *P.* *azotoformans* C2-114 showed the positive result for all direct PGP characteristics (Fig. 1) Twenty-two strains were capable of dissolving inorganic phosphate in the supernatant. Strain C2-114 was demonstrated the greatest content of available phosphorus (49.68 µg mL^−1^), which was significantly different (*p* < 0.05) from other isolates. Moreover, the maximum available potassium (1.28 µg mL^−1^) was measured in the cell-free supernatant of C2-114. l-tryptophan was added to TSB medium to produce IAA hormone. Isolate S81 produced 13.53 µg mL^−1^ of IAA, significantly more than other isolates. The amount of α-ketobutyrate hydrolyzed ACC which was one of by-products derived from the degradation of AAC substrate by ACC deaminase activity. We found that two isolates, S81 and R83, yielded the highest levels of α-ketobutyrate production in the range of 6.64–6.95 mM h^−1^ mg^−1^ protein. S81 was chosen because it produced the highest levels of ACC deaminase and IAA synthesis activity, and C2-114 was significantly capable of phosphate and potassium solubilization.

### Rhizobacterial identification and phylogenetic tree

**Figure 2:**
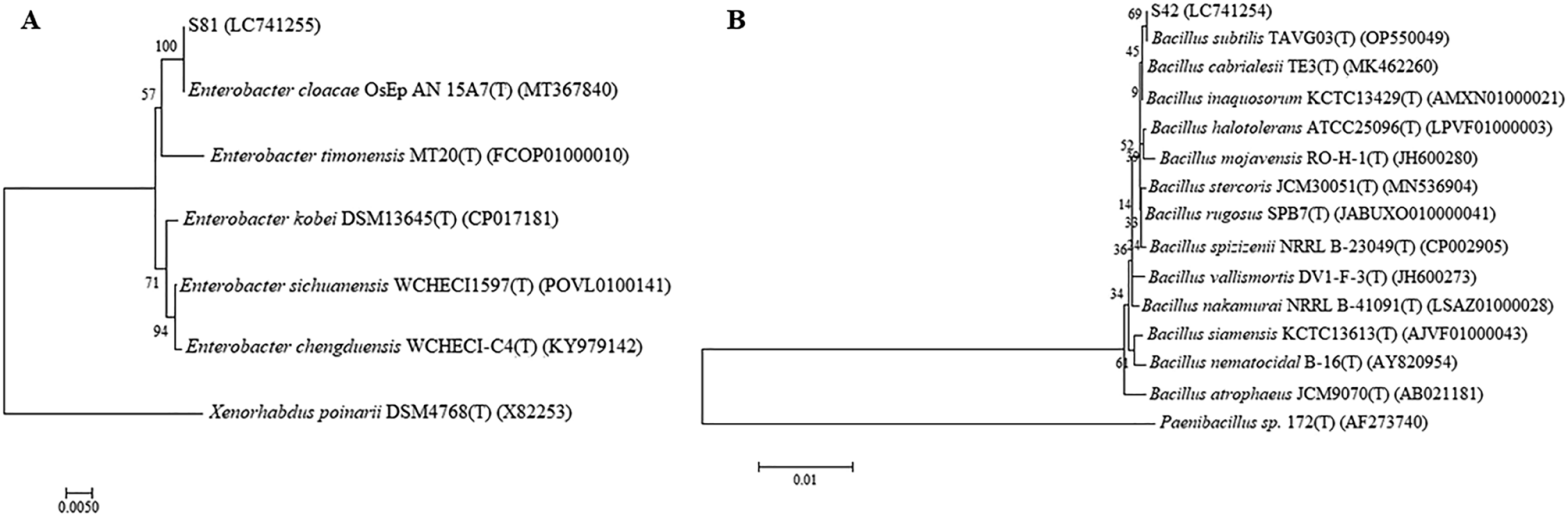
Phylogenetic tree based on 16S rDNA nucleotide sequences of S81 (**A**) and S42 (**B**) were constructed using the Neighbor-Joining with 1000 replicates of bootstrap. The bootstrap value is presented on the nodes. Maximum likelihood was used for computing the evolutionary distance.

The selected isolates S42 and S81 were identified based on the 16S rRNA gene sequences. The complete sequences of bacteria S42 and S81 matched the available sequences for *Bacillus* *subtilis* (100% identity) and *Enterobacter* *cloacae* (100% identity), respectively. In the phylogenetic analysis, S42 formed the solid clade with *Bacillus* *subtilis* type strain TAVG03^T^ (Fig. 2). Regarding S81, a clade belonging to *Enterobacter* *cloacae* type strain QsEp A&N 15A7^T^ was found (Fig. 2).

### Other PGP traits and colonization

**Figure 3:**
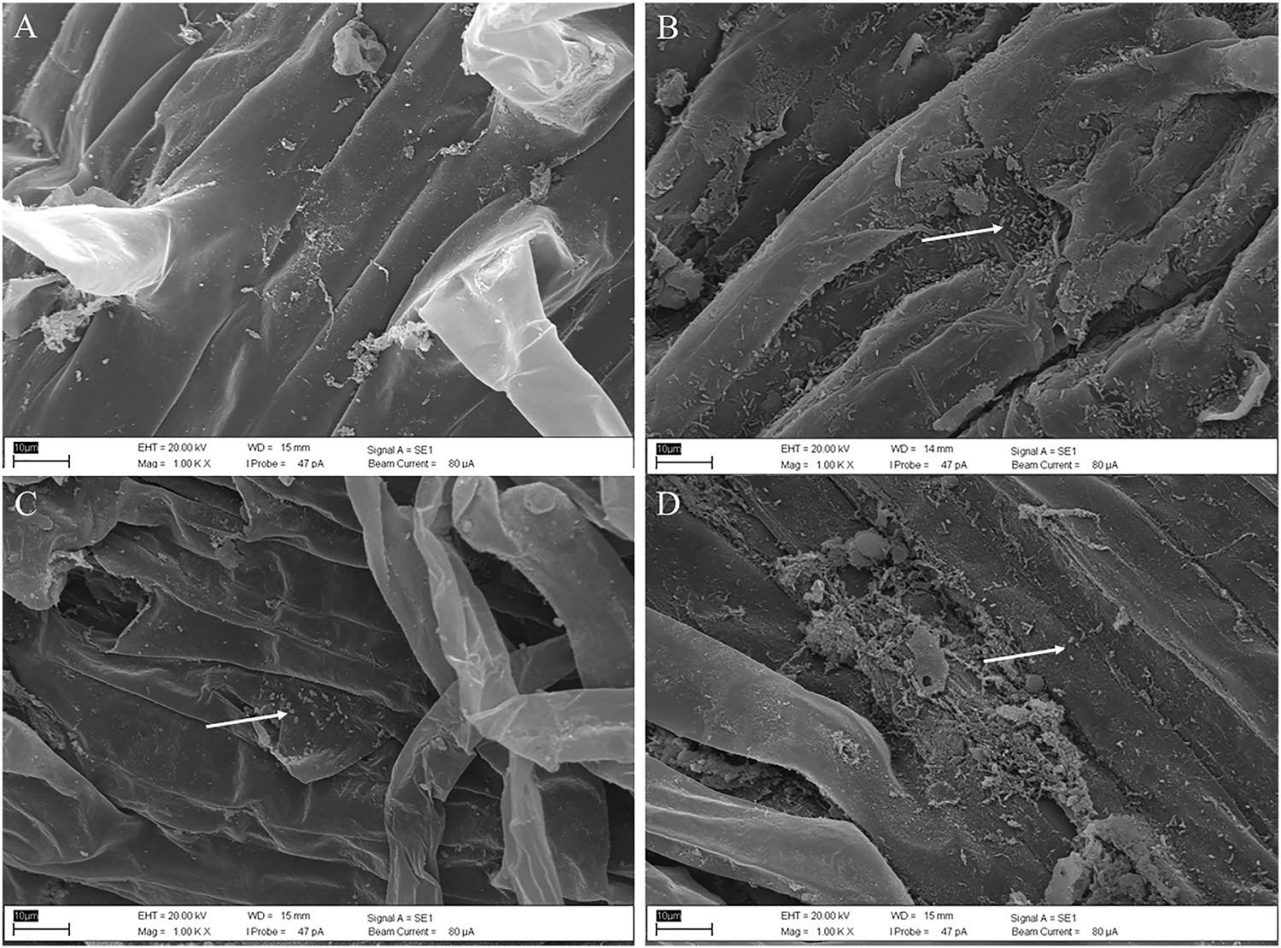
Scanning electron micrograph of Jerusalem artichoke roots. Clustered cells are presented by white arrowheads. (**A**) Control, (**B**) plant inoculated with *B.* *subtilis* S42, (**C**) plant inoculated with *E.* *cloacae* S81, (**D**) plant inoculated with *P.* *azotoformans* C2-114.

S42, S81, and C2-114 were examined for other PGP abilities: motility, and tolerance to concentrations of NaCl and PEG (Table 2). S42 inhibited the growth of *S.* *rolfsii,* whereas the other two PGP isolates were incapable of pathogen antagonism. S81 and C2-114 demonstrated multiple actions including siderophore and ammonia production, oxalate oxidase, and polyamine synthesis. Three strains proved capable of producing EPS and biofilm. C2-114 presented greatest tolerance to abiotic stresses such as salts, drought, and high temperature. S42, S81, and C2-114 were capable of motility, with S42 exhibiting the largest diameter among those examined. There was evidence that all selected strains possess indirect PGP characteristics and abiotic tolerance. The scanning electron microscope (SEM) revealed the colonization effectiveness of Jerusalem artichoke roots (Fig. 3). Our results showed the root surface was colonized by bacterial cells. Cells of three strains were distributed in the vicinity of the epidermal rough surface.

**Table 2:** Other PGP characteristics and tolerance to stress environment of selective bacteria.

Tests	S42	S81	C2-114
*S.* *rolfsii* inhibition growth	49.0 ± 1.7	−	−
Siderophore	−	+	+
Ammonia production	−	+	+
Oxalate oxidate enzyme	+	+	+
Catalase activity	+	+	+
Oxidase activity	+	+	+
Polyamine	−	+	+
Exopolysaccharide	+	+	+
Biofilm	+	+	+
Motility:Swimming	8.5	8.5	3.0
Motility:Swarming	8.5	0.8	0.6
Motility:Twitching	8.5	0.8	0.6
Growth at 40 °C	+	+	+
NaCl tolerance 3% (w/v)	+	+	+
NaCl tolerance 6% (w/v)	−	+	+
NaCl tolerance 9% (w/v)	−	−	+
PEG tolerance 20% (w/v)	−	+	+

Inhibition of *S.* *rolfsii* growth presented percentage of inhibition (%); Motility presented diameter (cm); − negative result; + positive result.

### Pot experiment

**Figure 4:**
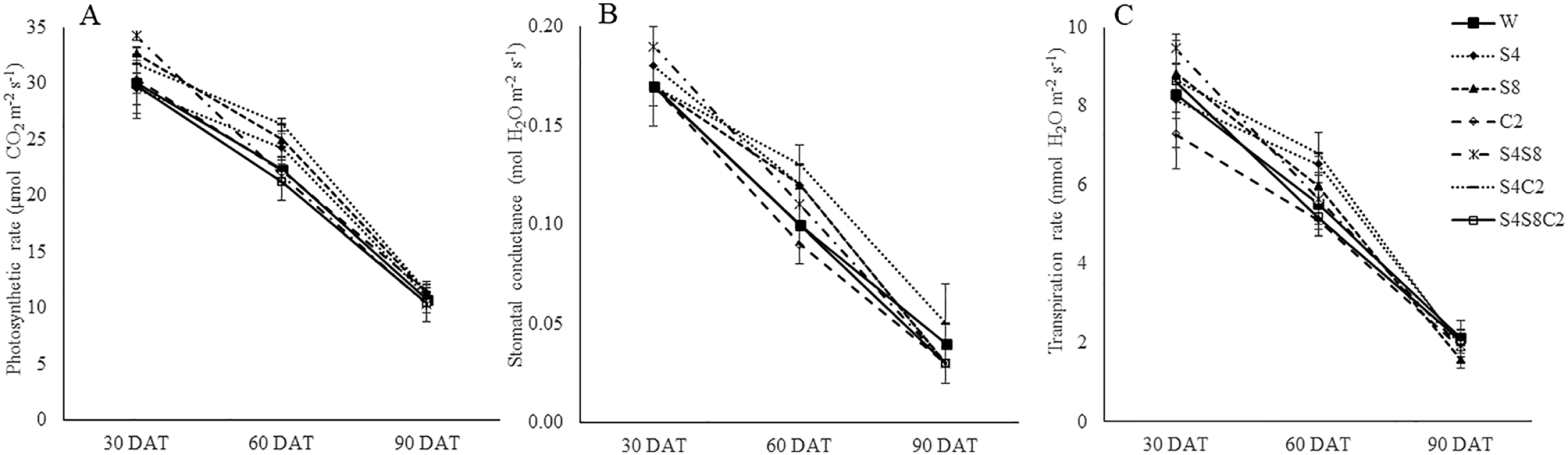
Effect of bacterial inoculation on photosynthesis parameters including photosynthetic rate (**A**), stomatal conductance (**B**), and transpiration rate (**C**). W: uninoculated plant as control, S4: *B.* *subtilis* S42, S8*:* *E.* *cloacae* S81, C2: *P.* *azotoformans* C2-114, S4S8: co-inoculation *B.* *subtilis* S42 and *E.* *cloacae* S81, S4C2: co-inoculation *B.* *subtilis* S42 and *P.* *azotoformans* C2-114, S4S8C2: mix of all strains.

**Figure 5:**
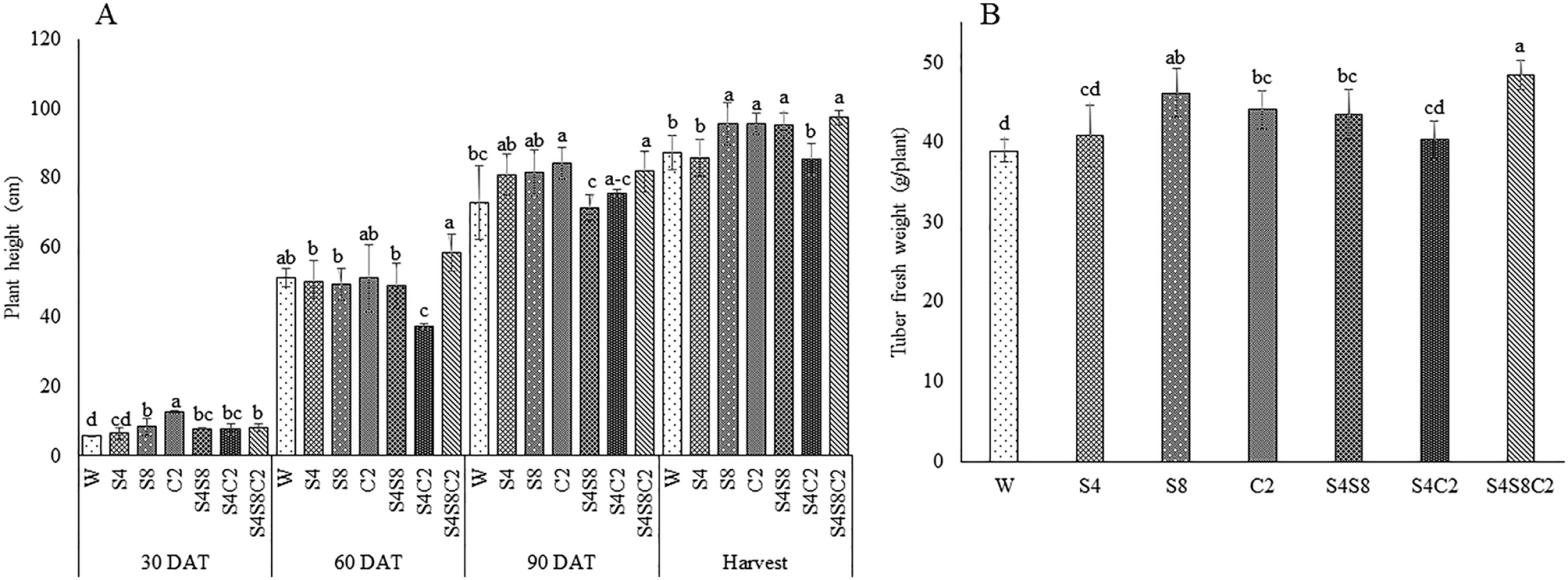
Effect of bacterial inoculation on plant height (**A**) and tuber fresh weight (**B**). Different letters represent significant differences (*p* < 0.05) according to the LSD test. W: uninoculated plant as control, S4: *B.* *subtilis* S42, S8*:* *E.* *cloacae* S81, C2: *P.* *azotoformans* C2-114, S4S8: co-inoculation *B.* *subtilis* S42 and *E.* *cloacae* S81, S4C2: co-inoculation *B.* *subtilis* S42 and *P.* *azotoformans* C2-114, S4S8C2: mix of all strains.

**Figure 6:**
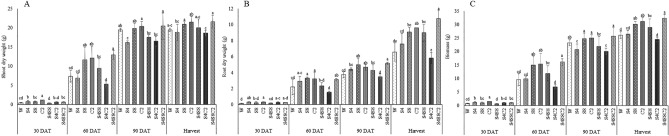
Effect of bacterial inoculation on shoot dry weight (**A**), root dry weight (**B**), and biomass (**C**). Different letters represent significant differences (*p* < 0.05) according to the LSD test. W: uninoculated plant as control, S4: *B.* *subtilis* S42, S8*:* *E.* *cloacae* S81, C2: *P.* *azotoformans* C2-114, S4S8: co-inoculation *B.* *subtilis* S42 and *E.* *cloacae* S81, S4C2: co-inoculation *B.* *subtilis* S42 and *P.* *azotoformans* C2-114, S4S8C2: mix of all strains.

**Figure 7:**
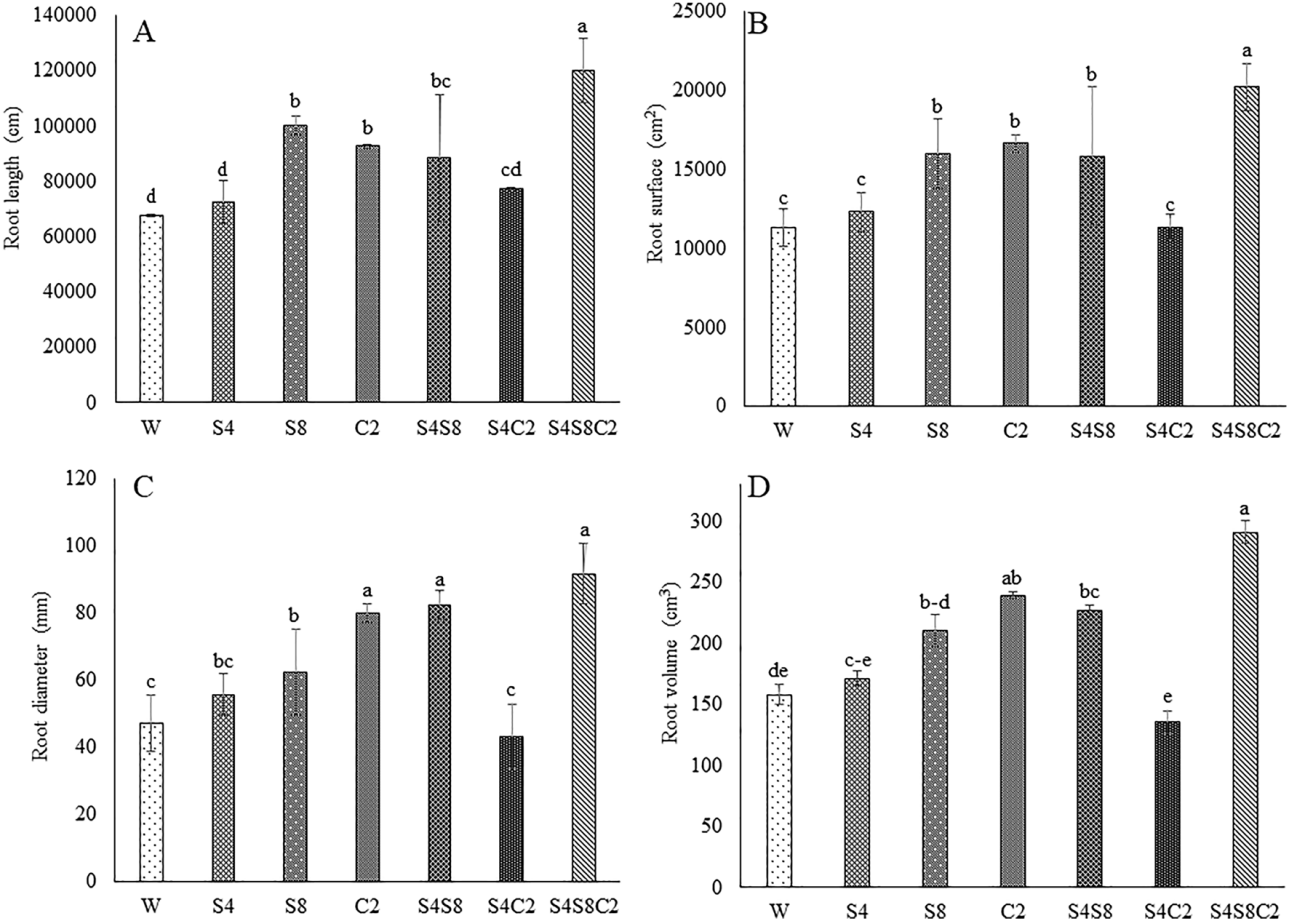
Effect of bacterial inoculation on root length, root surface, root diameter, and root volume. Different letters represent significant differences (*p* < 0.05) according to the LSD test. W: uninoculated plant as control, S4: *B.* *subtilis* S42, S8*:* *E.* *cloacae* S81, C2: *P.* *azotoformans* C2-114, S4S8: co-inoculation *B.* *subtilis* S42 and *E.* *cloacae* S81, S4C2: co-inoculation *B.* *subtilis* S42 and *P.* *azotoformans* C2-114, S4S8C2: mix of all strains.

Selected isolates such as S42, S81, and C2-114 were examined for biocompatibility. The results revealed no growth inhibition zone at intersection streaking. This finding indicated that the isolates could be mixed for plant inoculation. The photosynthesis rate (Pn), stomatal conductance (Sc), and transpiration rate (Tr) were monitored at 30, 60, and 90 DAT to assess the effects of PGP bacteria (Fig. 4). There was a reduction in the photosynthetic rate, stomatal conductance, and transpiration rate both treated with bacteria and untreated. The selected isolates had different effects on plant height and tuber fresh weight (Fig. 5). The consortium treatments (S42, S81, C2-114) significantly increased (p < 0.05) plant height (11.6%), shoot dry weight (10.8%), root dry weight (63.8%), and biomass (24.4%) (Fig. 6) when compared with control. Single inoculum treatment (S81 and C2-114) had the effect of increasing plant height and biomass at 60 DAT until harvesting time. However, S81 and C2-114 did not yield any improvement in root length and root surface, whereas mixed inoculums positively affected root length, root diameter, root surface, and root volume (Fig. 7). Moreover, the consortium treatment significantly increased tuber fresh weight in 24.2%.

## Discussion

Rhizobacteria that possess multiple PGP traits can be utilized for agricultural application.

The aim of this work was to screen rhizobacteria with a variety of direct and indirect PGP properties for stimulating the growth of Jerusalem artichoke. The data suggested that PGP consortium isolates (S42, S81, and C2-114) had significantly increased the development of Jerusalem artichoke by enhancing plant biomass and tuber fresh weight.

Interestingly, according to our observation of PGP and enzyme properties, some PGP isolates possessed direct PGP multifunctional traits, yet all hydrolase productions of indirect PGP traits were not consistently found. It is possible that some bacteria might not necessarily require the production of the enzyme in order to synthesize the nutrients. However, the best of our best knowledge, the common PGP and hydrolytic enzyme characteristics of bacteria isolated from the Jerusalem artichoke rhizosphere were recognized and examined for the first time. Rhizobacterial isolates produced hydrolase production, including cellulase, β-glucosidase, protease, phosphatase, and urease enzymes. These enzymes played the important role in the C-cycle, N-cycle, and P-cycle in the soils. Cellulose is the most abundant component of organic amendments. It is a main precursor for the synthesis of organic matter and organic carbon^15,18^. Moreover, these enzymes can also hydrolyze the cell walls of pathogens in order to protect the host plants^2^. Our results agreed with El-Deeb et al.^19^, Hazarika et al.^14^ and Magotra et al.^20^, who reported that *Bacillus* exhibited the highest levels of hydrolytic enzyme activities which could use for characterizing PGP candidates^12,14,19–24^.

The root plant absorbs phosphorus and potassium in soluble form to be utilized in growth^14,25^. Our findings in this study suggest that many isolates could solubilize both inorganic phosphate and potassium. The capacity of bacteria to solubilize phosphate and potassium was demonstrated by their utilization and conversion of sugar to organic acid, which lowered the pH of the media^26–28^. Moreover, several studied found that EPS-producing bacteria was able to support the high efficiency of solubilization^27,28^. Our isolate C2-114 was showed the ability to produce EPS, indicating that EPS enhances the efficiency of inorganic solubilization. Furthermore, it is possible for the P and K solubilizer to release zinc and silicate sources through the same mechanisms^22,23,28^.

IAA has the direct effect on the root elongation and indirect effect on improving of water and nutrient uptake efficiency^28^, respectively. The excreting of IAA in soils differs from those in vitro, depending on various factors. In a previous study by Sritongon et al.^29^, the same strain of *P.* *azotoformans *produced 78.75 µg mL^−1^ of IAA in the presence of l-tryptophan. However, varying amounts of IAA in vitro depend on several factors including the culture conditions, strains of species, and tryptophan concentrations^30^. *E.* *cloacae* produced the greatest amount of IAA. Our results are in agreement with the study by Kavamura et al.^12^, which revealed high concentrations of IAA produced by members of the *Enterobacteriaceae* family.

Plant growth is regulated by the interaction between IAA and ACC levels^16,31^. Our findings suggest that S81 produces the highest amount of IAA and ACC deaminase. The high concentration of IAA in the plant roots influences the ACC precursor, resulting in the formation of increased ethylene^31^. Excessive ethylene is the triggering signal with which plant growth is inhibited. ACC deaminases that are produced by PGP rhizobacteria hydrolyze the precursor of ethylene into α-ketobutyrate and ammonia to decrease ethylene^31–33^ Under normal and water-limited conditions, both IAA and ACC deaminase as the signal particularly enhances length, diameter, and volume of sunchoke root^16^. According to the research of Khan et al.^34^, both IAA and ACC deaminase showed the effect on increasing of plant biomass can increase plant biomass. ACC deaminase showed outstanding activity over others in stress condition. However, under normal conditions, ACC deaminase activity was greater than under stress^35^. ACC deaminase was required for PGP rhizobacteria to efficiently promote plant development under both conditions.

Three strains (S42, S81 and C2-114) were examined for various actions that could indirectly promote the plant. They presented positive results for secretion of oxalate oxidase, which leads to the destruction of phytopathogens such as *Sclerotinia* *sclerotiorum *and *Sclerotium* *rolfsii*^36^. S81 and C2-114 produced the siderophore, a chelator that scavenges ferric in environment soil. Iron is a necessary co-factor for metabolic enzymes, thus causing competition that suppresses plant pathogen growth^37^. However, we did not find an interaction between the siderophore and the inhibition of *S.* *rolfsii* growth. S42 revealed positive inhibition of *S.* *rolfsii*, which may have been caused by either protease cellulase, amylase, or the other hydrolase, in addition to bioactive compounds. The enzymes produced were part of a triggering role to protect the host plant. Moreover, hydrolases break the cell walls of plant pathogenic fungus which infect Jerusalem artichoke; these pathogens include *Fusarium* *oxysporum*, *Colletotrichum* *capsici*, and *S.* *rolfsii*^2,17^.

Two PGP strains gave a positive result of polyamine production. Polyamine (putrescine, spermidine, and spermine) improves plant development. Besides, it alleviates plants in adverse environments such as metal toxicity, oxidation, drought, salinity, and cold^38^. Three strains were capable of producing EPS and biofilm. They retain moisture on the surface of their cells to occupy and protect the root surface from dehydration, hence reducing the impacts of variable dryness, salinity and temperature^12^. *Bacillus* ap. and *Pantoea* sp. were the most prolific EPS producers^12,16,23^. Microbial biofilm increases the efficacy of microbe-root connection^14,23,24,39^, reduces infection from plant pathogen, and defends plants from stress^23,39^. It was proved that our selective strains could survive environmental stresses such as high temperature, salt, and drought. Microbes employed cellulase and pectinase to hydrolyze root cell wall and colonize the root tips and roots branch at the interior and exterior of the roots^19,30^. Our findings also showed that three strains displayed motility, such as swimming, swarming, and twitching. Selected strains are therefore candidates for strain possessing multiple functions in PGP, colonization, and survival in environmental soils.

PGP rhizobacteria activity in soil is not driven by a single species but by numerous species operating simultaneously^12,40^. The absence of an inhibitory zone at the intersection of perpendicular streaks indicated that the hydrolase-producing strain and PGP strains were compatible. It was possible to combine strains and use them to promote the growth of Jerusalem artichoke. Thus, they were conducted in a non-sterile soil meaning their performance may differ from that exhibited in vitro.

According to the physiological of plant results, the photosynthetic rate, stomatal conductance, and transpiration rate were showed the decreasing trends from 30 to 90 DAT. Stomatal conductance and transpiration responded to preventing water loss in water deficit situations^6^. Significant differences were observed in photosynthetic rate during drought stress^1,16^. These parameters indicated variable responses to stress.

In this case, the height of plant, shoot dry mass, root dry mass and total biomass were increased when it treated with a single inoculant of bacterial isolate C2-114. Similarly, to Sritongon et al.^29^, C2-114 increased in sunchoke biomass at initial growth, which showed a strong correlation with IAA. In addition, enhanced root elongation, diameter, and surface were regarded as positive effects of S81 and C2-114 inoculation. This may be the result of the presence of IAA and ACC deaminase. ACC and IAA were cooperate to modulate the ratio of ACC to IAA in modified root systems^16,27,34^.

The consortium of selected isolates revealed a significant difference in their capacity to increase height, shoot dry weight, root dry weight, biomass at 60 DAT to harvest and tuber fresh weight. Similar to Hazarika et al.^14^ PGP rhizobacteria consortia had more effect on plants than individual inoculum. Consortia of bacterial isolates alleviated the negative effect of salinity stress and increased biomass of French bean^27^. Our data indicates that mixed culture seems to contribute to synergetic activity by enhancing the Jerusalem artichoke. Therefore, a consortium of both PGP (two strains) and hydrolase producing rhizobacteria could improve plant biomass and tuber productivity better than a single inoculant via multiple direct and indirect PGP activity as well as extracellular enzyme synthesis. Moreover, applying the mixed inoculant showed higher number of survival cells in their natural environments than using a single inoculant^12,16,40–42^. It can be utilized as bio-fertilizer in sunchoke. However, this work has limitations which potential consortium isolates need to be confirmed in the field conditions. For further research, we will focus on the efficacy of field-applied organic amendments in promoting plant growth for the development of biofertilizers.

## Conclusion

Our study revealed rhizobacteria possess multifaceted PGP direct and indirect activities that supported their capacity to enhance plant productivity. Two PGP strains (*Enterobacter* *cloacae* and *Pseudomonas* *azotoformans*) and hydrolase producing strain (*Bacillus* *subtilis*) were confirmed to enhance the growth of plants. These interesting results suggested that the mixed three isolates stimulated the growth of plants such as the biomass and the tuber of Jerusalem artichoke. Thus, this consortium demonstrated high potential for plant growth promotion. In order to verify soil improvement; the consortium of inoculants can be used as a supplementation of soil amendment or compost and developed to the bio-fertilizer product and used it infertile soils for sustainable improvement.

## Materials and methods

### Plant materials and rhizobacterial isolation

Samples of Jerusalem artichoke roots with adhering soil were collected from 0 to 10 cm depths at farm of the field crop department, faculty of Agriculture, Khon Kaen University, Khon Kaen Thailand (16°47′45′′N, 102°81′07′′E). The roots were stored in an ice box before being transferred to the laboratory. The root of each sample was cut into approximately 0.5 cm segments. Five grams of root segments were transferred to 45 mL of diluent (0.85% (w/v) NaCl and 0.1% (w/v) tween 20) in an Erlenmeyer flask. All flasks were shaken at 180 rpm for 1 h.0.0.1 mL aliquot of tenfold serial dilution was spread on soil extract medium^43^: 1.0 g peptone, 1.0 g yeast extract, 0.5 g K_2_HPO_4_, 0.5 g (NH_4_)_2_SO_4_, 0.05 g MgSO_4_·7H_2_O, 0.01 g FeCl_3_, 0.1 g CaCl_2_, 15.0 g agar, 250 mL soil extract, 15.0 g agar, and 750 mL of distilled water and R2A medium: 0.5 g casein hydrolysate, 0.5 g proteose peptone, 0.5 g glucose, 0.5 g soluble starch, 0.3 g K_2_HPO_4,_ 0.024 g MgSO_4_·7H_2_O, 0.3 g sodium pyruvate, 15.0 g agar, and 1000 mL of distilled water. The plates were incubated at 30 °C for 48 h. Different bacterial colonies based on morphology were selected and purified using the cross-streaking technique on a Petri dish containing tryptic soy agar (TSA) (Himedia). Bacterial isolates were stored in 20% glycerol solution at − 20 °C until further use.

### Screening of plant growth-promoting (PGP) traits

All isolates were screened for PGP traits such as nitrogen fixation, phosphate solubilization, potassium solubilization, synthesis of indole3-acetic acid and 1-aminocyclopropane-1-carboxylic acid (ACC) deaminase activity. Regarding nitrogen fixation, each isolate was inoculated into Ashby’s nitrogen free broth^44^. NBRIP agar^45^ and Aleksandrov agar^46^ were used to evaluate the solubilization of phosphate and potassium, respectively. Each isolate was cultured in tryptic soy broth (TSB) (Himedia) supplemented with 1 g L^−1^ l-tryptophan (Acros Organics) for 24 h, and Salkowski’s reagent added^47^. ACC deaminase activity was screened on DF minimal salt agar^48^ with 3 mM of 1-aminocyclopropane-1-carboxylic acid (ACC) instead of ammonium sulfate^32^. *Pseudomonas* *azotoformans* C2-114 (Accession number LC130639), which possessed nitrogen fixation, phosphate and potassium solubilization, IAA production, and siderophore traits^29^ was used to investigate ACC deaminase activity and other PGP and hydrolase activities.

### Quantification of PGP activities

#### Phosphate solubilization

Phosphate solubilization of each PGP isolate was determined using NBRIP medium^45^. PGP isolate was grown in TSB medium at 30 °C, 150 rpm for 24 h. The bacterial cell (consisting of approximately 10^8^ CFU mL^−1^ as inoculum (100 µL) of each isolate was transferred to a 5 mL sterile NBRIP broth tube containing 0.5% (w/v) tricalcium phosphate powder as the insoluble phosphate. Tubes were done in triplicate, and incubated at 30 °C, 150 rpm for 7 days. Aliquots were of 1 mL supernatant of each sample and control, 1 mL of molybdate-vanadate reagent^49^. All tubes were incubated in darkness for 20 min. The development of a yellow color was used to quantify soluble phosphorus. The sample was measured at 420 nm (U-5100, Hitachi). A standard curve was constructed using KH_2_PO_4._

#### Potassium solubilization

Potassium solubilization of each PGP isolate was determined using Aleksandrov medium^46^. Inoculum (100 µL) was transferred to 5 mL of sterile Aleksandrov broth containing 0.2% (w/v) potassium aluminium silicate powder (Himedia) as an insoluble potassium source. The tubes were done triplicate and incubated at 30 °C, 150 rpm for 7 days. Aleksandrov broth medium without inoculation served as the control. Soluble potassium in the supernatant was measured at 776.5 nm using an atomic absorption spectrometer (AAnalyst 100, Perkin Elmer) with flame air C_2_H_2_ and a slit of 0.7 nm. A standard curve was constructed using KCl.

#### Indole-3-acetic acid (IAA) production

IAA production of each PGP isolate was determined using Salkowski’s reagent^47^. Inoculum (100 µL) of each isolate was added into 5 mL sterile TSB medium supplemented with 1 g L^−1^ l-tryptophan (Acros Organics). The tubes were done in triplicate and then incubated at 30 °C, 150 rpm for 48 h. Aliquots of 1 mL of supernatant were mixed with 2 mL of Salkowski’s reagent^47^, before being incubated in the darkness for 30 min. The appearance of red coloration was used to quantify IAA production. The sample was read at 530 nm. A standard curve was constructed using IAA (Acros Organics).

#### ACC deaminase activity

ACC deaminase activity was determined according to Penrose and Glick^32^. The isolates were cultured in 5 mL of DF minimal salt broth medium^48^ with an addition of 3 mM of ACC (Acros Organics), and then incubated at 30 °C, 150 rpm, for 72 h. The cell was harvested by centrifugation, then washed with 0.1 M Tris–HCl (pH 7.5) and resuspended in 600 µL of 0.1 M Tris–HCl (pH 8.5). Toluene (30 µL) was added into cell solution and vortexed. To determine the activity of the ACC deaminase, toluenized cells in an aliquot of 200 µL were pipetted into a 1.5 mL sterile microcentrifuge tube, mixed with 20 µL 0.5 M ACC and 200 µL of 0.1 M Tris–HCl (pH 8.5). The mixture was then incubated at 30 °C for 30 min. 1 mL of 0.56 N HCl was added, vortexed. Aliquot of the supernatant (1 mL) was mixed with 800 µL of 0.56 N HCl and 300 µL of the 0.2% (w/v) 2,4 dinitrophenylhydrazine reagent (dissolved in 2 N HCl), respectively, before incubation at 30 °C for 30 min. 1 mL of 2 N NaOH was then added and thoroughly mixed prior to being measured at 540 nm. Standard curve was constructed using α-ketobutyrate (Sigma-Aldrich). Soluble protein was performed according to Bradford assay^50^. In order to create a standard protein curve, bovine serum albumin (Acros Organics) was used.

### Screening of hydrolytic enzymes activities

All isolates were examined for cellulase, β-glucosidase, α-amylase, protease, urease, and phosphatase traits. For evaluation, secreted cellulase and β-glucosidase were tested on solid minimal medium^51^: 6.8 g Na_2_HPO_4_, 3.0 g KH_2_HPO_4_, 0.5 g NaCl, 1.3 g (NH_4_)_2_SO_4_, 0.5 g MgSO_4_·7H_2_O, 15.0 g agar, 1000 mL distilled water, to which was added 0.5% (w/v) cellulose for cellulose hydrolysis and 0.1 mL of 0.04% (w/v) 4-methylumbelliferyl-β-d-glucopyranoside (MUG) (Acros Organics) to spread on the agar before inoculation. After incubation at 30 °C for 48 h, Gram’s iodine solution was used to evaluate cellulose hydrolysis. Fluorescent halo of MUG hydrolysis appeared under UV light. For the assessment of proteolytic production, each isolate was inoculated on skim milk agar (Peptone 5 g, beef extract 3 g, skim milk 10 g, agar 15 g—1000 mL). A clear zone was visible around the colony after incubation at 30 °C for 48 h. To evaluate α-amylase, production was performed on starch agar (Peptone 5 g, beef extract 3 g, starch 10 g, agar 15 g—1000 mL). Starch hydrolysis was examined using Gram’s iodine solution. For phosphatase production, the culture was spotted on the TSA medium containing 0.01% (w/v) phenolphthalein bisphosphate tetrasodium salt (Sigma-Aldrich), examined by 8.4% (v/v) ammonium hydroxide^26^. Urease production was carried out on Christensen’s medium urea agar (Himedia) containing sterile 2% w/v urea. The colony was observed displaying a pinkish-red color on the agar^52^.

### Other PGP, motility, and stress tolerance

Selected isolates were examined for indirect PGP traits including siderophore production, antagonistic test, polyamine production, ammonia production, oxalate oxidase enzyme production, biofilm, exopolysaccharide production and the microbial characteristic under environmental stress conditions. Chrome Azurol S (CAS) agar was used to evaluate siderophore formation^53^. The suppression of *Sclerotium* *rolfsii* was investigated following the dual method described in Sritongon et al.^29^. The percentage of inhibition was calculated from center to the edge of fungal mycelia length: (A − B)/A × 100 (A: Control, B: bacterial inoculation). Moeller’s decarboxylase broth base (Himedia) pH 7.0 supplemented with 1% (w/v) l-arginine hydrochloride (Acros Organics) was used to test polyamine production. Ammonia production was evaluated using 4% (w/v) peptone water and examined with Nessler’s reagent^54^. Oxalate oxidase was tested on the agar plate containing potassium oxalate as the sole carbon source^55^. Motility assay that included swimming, swarming, and twitching was carried out using nutrient medium (0.5% (w/v) peptone and 0.3% (w/v) beef extract) having 0.3%, 0.5%, and 1.0% (w/v) agar, respectively. Biofilm production was performed using crystal violet staining with a modified version of Latorre et al.^56^. Exopolysaccharide (EPS) production was performed in accordance with Kavamura et al.^12^ by replacing saccharose with 10% (w/v) glucose. The capability of isolates to tolerate a stressful environment was tested by culturing in nutrient broth with varied NaCl concentration, 20% (w/v), polyethylene glycol (PEG6000), and growth at 40 °C.

### 16S rRNA identification and phylogenetic analysis

Selective rhizobacteria was cultured in nutrient broth on a rotary shaker at 150 rpm and 30 °C for 18 h. The genomic DNA was extracted according to the manufacturer’s protocol using the TIANAMP bacterial DNA kit (Tiangen biotech, China). 16S rRNA gene was amplified with the universal primers, 8F: 5′ AGA GTT TGA TCM TGG CTC AG 3′^57^ and 1512R: 5′ ACG GYT ACC TTG TTA CGA CTT 3′^58^. The reaction was carried out on a FlexCycler2 PCR thermal cycler (Analytik Jena, Germany) using the following program: initial denaturation at 95 °C, 10 min; 35 cycles of denaturation at 94 °C, 1 min; annealing at 55 °C, 1 min; extension at 72 °C, 90 s, and final extension at 72 °C, 10 min. The PCR product was checked on 1.5% (w/v) agarose gel, prior to being submitted for purification and sequencing by the 1st BASE Laboratories Sdn. Bhd., Malaysia. The nucleotide sequences were subjected to BioEdit program. Complete sequences were analyzed by using the BLASTN to compare with strain types in GenBank database. The ClustalW in MEGA program 7.0.0 was aligned and a phylogenetic tree was constructed using neighbor-joining method including 1000 bootstrap replicates.

### Root colonization and scanning electron microscope (SEM) analysis

The Jerusalem artichoke seedling genotypes HEL65 was obtained from Agricultural Faculty, Khon Kaen University. For plant preparation, a tuber piece having two to three buds of the sunchoke variety HEL65 were germinated in a box containing moist coconut peat. Then, they were transferred to charred rice husk and soil (1:1 w/w) on a plastic tray with 4 cm diameter wells and cultivated until two true leaves expanded^59^. The plants were collected and washed with tap water to remove the charred rice husk and soil. The plant roots were twice rinsed with sterilized distilled water. The root system of each plant was dipped gently into a 15 mL glass tube containing 10 mL sterile distilled water. Then, bacterial suspension (consisted of approximately 10^8^ CFU mL^−1^ selected isolate was transferred to the tube. The control treatment consisted of roots in sterilized distilled water. All tubes were incubated at room temperature for 24 h. To prepare the sample for scanning electron microscope (SEM) analysis, the whole root of each plant was rinsed twice with sterile 0.1 M phosphate buffered saline (PBS) (pH 7.2). The root was next soaked in 2.5% (v/v) glutaraldehyde at 4 °C for 2 h, then segmented between 3–5 mm and immersed in PBS for 10 min, three times. To dehydrate the samples, they were immersed in a series of ethanol dilutions (50, 60, 70, 80, and 90% v/v) for 15 min at each gradient, and twice absolute ethanol at terminal concentration for 30 min, before ethanol removal. The samples were dried with a critical point drier (CPD 7501, Polaron), then attached to aluminum stubs with double-coated carbon tape and gold-coated in a sputtering coating machine (108 auto, Cressington). A Leo 1450VP scanning electron microscope was employed to identify the strains that colonized on the root.

### Biocompatibility test

The biocompatibility of selective isolates was evaluated using the perpendicular streaking technique^41^. Each isolate was simultaneously streaked across the center culture in perpendicular on TSA medium. The plates were incubated at 30 °C for 24 h. Bacterial isolates were able to grow at intersection of each culture, indicating bio-compatibility.

### Pot experiment

The enhancement of Sunchoke growth by selected rhizobacteria isolates was performed in an open-sided greenhouse at the Field Crop department, Faculty of Agriculture, Khon Kaen University, Thailand (16°47′59′′N, 102°81′61′E). The soil was air-dried, passed through a 2-mm sieve, and then added to a pot (15 inches in diameter and 11 inches in depth) to fill approximately 21 kg. The soil was classified as sandy loam with pH 6.57 and electrical conductivity of 0.63 dS m^−1^. Soil chemical properties were organic carbon 0.20%, organic matter 0.35%, total nitrogen 0.02%, available phosphorus 12.00 mg kg^−1^, available potassium 24.50 mg kg^−1^, and cation exchange capacity 3.70 c mol kg^−1^.

A pot experiment was conducted in a randomized complete block design (RCBD) with four replicates. The pot experiment was performed during the early rainy season (April to September 2019). The experiment consisted of the following seven treatments: control without bacterial inoculation (W), inoculated with hydrolase-producing isolates (S4), inoculated with PGP isolate (S8), inoculated with PGP isolate (C2), inoculated with PGP isolate and hydrolase producing isolate (S4S8), inoculated with PGP isolate and hydrolase producing isolate (S4C2), inoculated with two PGP isolates and hydrolase producing isolate (S4S8C2). Jerusalem artichoke variety HEL65 was prepared as described above. All methods were performed in accordance with relevant guidelines and regulations. In each pot, one plant with two true leaves was transplanted and established at the same depth. Each isolate was individually cultured in TSB medium. The isolates were grown with agitation of 150 rpm at room temperature for 18 h. The bacterial cells were collected by centrifugation at 6000 rpm for 10 min, then washed twice with sterile distilled water. The cell pellets were resuspended in 0.85% (w/v) NaCl and adjusted to a concentration 10^9^ CFU mL^−1^. For co-inoculation and consortium inoculation, equivalent volumes of each isolate were mixed as the inocula. Bacterial inocula (20 mL) were inoculated using a syringe to the vicinity of the roots of the seedling.

### Plant analysis

Plant height, shoot and root dry weights were recorded at 30, 60, 90 days after transplanting (DAT) and harvest time (140 days). The shoot and root samples were separated. The roots were placed on a sieve and carefully rinsed with tap water to eliminate soil particles. Using a scanner (Perfection V800™ Photo, Epson), root morphology such as total length, surface area, volume, and average diameter was scanned and analyzed using Win-Rhizo Pro2004a software (REGENT Instruments Inc., Canada). The shoots and roots were dried in a hot air oven at 70 °C for 72 h for constant mass. The biomass of the plant was presented using the sum of the dried shoot and root mass. The photosynthetic parameters were measured from its true leaves using a portable photosynthesis system (LI-6400XT, LI-COR Bioscience, USA). Fresh weight of the tubers for each treatment was recorded at harvesting.

### Statistical analysis

The in vitro data and pot experiment were subjected to a one-way analysis of variance (ANOVA). All statistical analysis was performed using the Statistix 10 software. The mean of treatments of all tests was identified by least significant difference (LSD) test at *p* < 0.05.

## Data Availability

S42 and S81 DNA sequences have been deposited to the DDBJ with accession numbers LC741254 and LC741255, respectively. *Bacillus* *subtilis* S42 gene for 16S rRNA, partial sequence (https://www.ncbi.nlm.nih.gov/nuccore/LC741254). *Enterobacter* *cloacae* S81 gene for 16S rRNA, partial sequence (https://www.ncbi.nlm.nih.gov/nuccore/LC741255). All data in this study are available upon request from the corresponding author at nunrid@kku.ac.th.
